# Distribution and fractionation of rare earth elements in suspended particulate matter in a coastal river, Southeast China

**DOI:** 10.7717/peerj.12414

**Published:** 2021-10-29

**Authors:** Man Liu, Guilin Han

**Affiliations:** Institute of Earth Sciences, China University of Geosciences (Beijing), Beijing, China, China

**Keywords:** Lanthanides, REE fractionation, Suspended particulate matter, Weathering processes, Jiulongjiang River

## Abstract

**Background:**

In the river system, the geochemistry of rare earth elements (REEs, a series of elements from La to Lu) in suspended particulate matter (SPM) is generally controlled by rock weathering processes and hydrochemical characteristics, as well as being affected by anthropogenic activities. However, the variations of geochemical characteristics and behaviors of REEs in SPM with a salinity gradient from the inland river to the estuary have been short of a systematic understanding.

**Methods:**

The REE concentrations, Post Archean Australia Shale (PAAS)-normalized REE, La/Yb, La/Sm, and Sm/Yb ratios of SPM were investigated in the Jiulongjiang River, which is a coastal river mainly flowing through granite rocks in Southeast China. The correlation relationships between physicochemical parameters (including water pH, total dissolved solids (TDS), HCO_3_^–^ concentrations, and the concentrations of major elements of SPM) and PAAS-normalized REE ratios of SPM were analyzed to determine the factors that affect the REE concentration and fractionation of SPM in the different regions of Jiulongjiang River, including the main stream and tributary of Beixi River, Xixi River, Nanxi River, and estuary. Additionally, the Ce, Eu, and Gd anomalies of SPM were estimated.

**Results:**

The average ∑REE concentration of SPM (352 mg/kg) in the granite rock basin was twice higher than the mean value (175 mg/kg) of the world’s rivers. The PAAS-normalized REE ratios of SPM in the main rivers including Beixi River (main stream), Xixi River, and Nanxi River were near due to the same lithologic distribution. In the tributary of Beixi River, the input of low-weathered carbonate minerals which contain very few REE caused the lower REE concentrations of SPM. The PAAS-normalized REE ratios of SPM in the estuary were significantly lower than those in the main rivers, which was mainly attributed to the significant REE removal with the increment of salinity. The enrichment of LREE relative to HREE in SPM increased with decreasing water pH in the main rivers. In the estuary, the preferential removal of dissolved LREE occurred compared to HREE with the increment of salinity. The negative Ce and Eu anomalies of SPM occurred in both the main rivers and estuary region and rare Gd pollution was present in the basin. Additionally, human activities caused the increment of REE concentrations and more negative Ce anomaly at some specific sites, such as dam effect and agricultural pollution.

**Conclusions:**

The REE concentrations and fractionations of SPM in river water mainly depend on lithologic distribution and riverine pH, while they are affected by salinity in the estuary.

## Introduction

The rare earth elements (REEs, a series of lanthanides from _57_La to _71_Lu) have been widely concerned regarding the environmental influences such as occurrences, concentration levels, and transformation processes in river systems (Bayon et al., 2015; Elderfield, Upstill-Goddard & Sholkovitz, 1990; Xu & Han, 2009), estuary systems (Ma et al., 2019; Suja, Fernandes & Rao, 2017), coastal sea (Amalan et al., 2018; Elderfield, Upstill-Goddard & Sholkovitz, 1990), and urban runoff system (Petelet-Giraud, Klaver & Negrel, 2009; Shajib et al., 2020). Generally, the REEs occur coherently in natural environments due to similar chemical properties. According to the geochemical behaviors of REEs, the three groups including light REE (LREE, from La to Nd), middle REE (MREE, from Sm to Ho), and heavy REE (HREE, from Er to Lu) are commonly classified. The weathering of rocks is the primary source of REEs in the earth’s surface environments (Yang et al., 2021). However, as the important annexing agents of modern materials, REEs have been extensively applied in agriculture, military, aviation, and medical industries (Altomare, Young & Beazley, 2020; Bau & Dulski, 1996; Dushyantha et al., 2020; Louis et al., 2020; Volokh et al., 1990). Thus, distinguishing natural and anthropogenic REE sources is environmentally meaningful (Naccarato et al., 2020).

In the river system, REEs in rocks mobilize into riverine suspended particulate matter (SPM) and dissolved loads during the rock weathering processes. The two forms of REEs can be interconverted through the physical and chemical processes, such as adsorption, colloid flocculation, dissolution, and complexation (Brookins, 1989; Sholkovitz, Landing & Lewis, 1994; Xu & Han, 2009). Generally, the REEs of SPM (59–289 mg/kg in the world’s rivers) are far more than those in dissolved loads (13–2,484 µg/kg) (Goldstein & Jacobsen, 1988), for example, da Silva et al. (2018) reported that more than 95% of total riverine REEs were carried by SPM in the Ipojuca River. Rock weathering products and re-suspended sediments are the primary sources of SPM (Roussiez, Aubert & Heussner, 2013; Vercruysse, Grabowski & Rickson, 2017). In some sites with intensive human activities, a large part of REEs in SPM is likely attributed to anthropogenic sources (Bau & Dulski, 1996; Louis et al., 2020). Thus, the REEs of SPM in the river, even other water bodies, record the integrated information about the geochemical characteristics of REE in SPM controlled by weathering processes and human disturbances since the Anthropocene (Linders et al., 2018; Rogers et al., 2019).

The alterations of hydrochemical characteristics caused by the input of weathering products and anthropogenic activities can substantially affect the concentration and fractionation of REE in SPM (Jones et al., 2016; Smith & Liu, 2018). For example, water pH, redox potential, and organic matter concentration significantly influence the dissolution and enrichment of SPM-associated REE in an inland river (Goldstein & Jacobsen, 1988; Johannesson et al., 2004; Migaszewski, Galuszka & Dolegowska, 2019). However, the REE removal in the estuary region with the increment of salinity significantly reduces the REE concentration of SPM (Elderfield, Upstill-Goddard & Sholkovitz, 1990; Nozaki et al., 2000). On the other hand, the REE fractionation can cause the variational concentrations of different REEs (Louvat & Allegre, 1998), due to the alterations of water physicochemical properties (Elias et al., 2019; Michaelides et al., 2010). Generally, the fractionations of REEs in SPM are also closely related to the water physicochemical properties, such as water pH, salinity, and the colloids of Fe/Mn oxides/hydroxides (Dagg et al., 2004; Krickov et al., 2020; Quinn, Byrne & Schijf, 2006). For example, LREE is preferentially adsorbed by clay mineral particles compared to HREE, resulting in HREE depletion relative to LREE in SPM (Chelnokov, Bragin & Kharitonova, 2020). Xu & Han (2009) reported that MREE and Ce are preferentially adsorbed by Fe/Mn oxides/hydroxides, while other LREEs are easily absorbed by clay colloids. Compared to LREE, HREE is more easily separated with SPM into the dissolved load with increasing river water acidity (Migaszewski, Galuszka & Dolegowska, 2019). Elderfield, Upstill-Goddard & Sholkovitz (1990) reported that the preferential removal of LREE occurred with the increment of salinity in the estuary region. Additionally, the redox potential-dependent Ce anomaly (Alderton, Pearce & Potts, 1980), lithology-dependent Eu anomaly (Han et al., 2009), and anthropogenic pollution-dependent Gd anomaly (Bau & Dulski, 1996; Louis et al., 2020) are the useful indexes of REE fractionation. Overall, the geochemical characteristics (including concentration and fractionation) of REEs in SPM can provide much useful information about weathering processes, hydrochemical characteristics, and anthropogenic activities (Cholet et al., 2019; Smith & Liu, 2018).

REEs of SPM are useful environmental tracers to indicate weathering processes and anthropogenic activities. The Jiulongjiang River, which is a coastal river mainly flowing through granite rocks in Southeast China (Liu & Han, 2020), is a perfect area to study the variations of geochemical characteristics and behaviors of REEs in SPM with a salinity gradient from the inland river to the estuary. Thus, the concentration and fractionation of REEs in SPM in river and estuary were investigated and the effects of weathering processes and anthropogenic activities on them were analyzed in the present study. This study aims to: (1) investigate the concentrations and fractionations of REEs in SPM in river and estuary, (2) determine their controlling factors from weathering processes and anthropogenic activities, and (3) understand the differences in the concentrations and fractionations of REEs in SPM between in river and estuary.

## Materials and Methods

### Study area

The Jiulongjiang River (24°05′N–25°55′N, 106°50′E–118°20′E), which is situated at the southeast coastal margin of the Chinese mainland, has the basin area of about 14,740 km^2^ (Liu & Han, 2020). The main stream flows across the Longyan and Zhangzhou cities with a channel length of 260 km and an annual water discharge of 14 km^3^. The Jiulongjiang River is comprised of the three main rivers, including Beixi River, Nanxi River, and Xixi River. The basin is mainly controlled by the sub-tropic monsoon climate. The period with the majority of rainfalls (∼80%) and the highest air temperature are simultaneously concentrated in the wet season from April to September. In the basin, mean annual precipitation (MAP) ranges from 1400 mm to 1800 mm and the mean annual temperature (MAT) varies from 19.9 °C to 21.1 °C (Liu & Han, 2020). Magmatic rocks and clastic sedimentary rocks are widely distributed in the basin, while metamorphic rocks and limestones are exposed rarely (Li, Qiu & Yang, 2014). The red soils, which are classified into Alisols (WRB, 2014), account for >90% area of the basin. The region of upper reaches mainly flows through mountain areas with high forest coverage and slight agricultural and industrial perturbations, while other regions are affected by different degrees of human activities (Huang et al., 2013). Furthermore, the Wananxi Reservoir regulates the discharge of river water in the upper reaches of the Beixi River.

### Sample collection and in-field measurements

**Figure 1:**
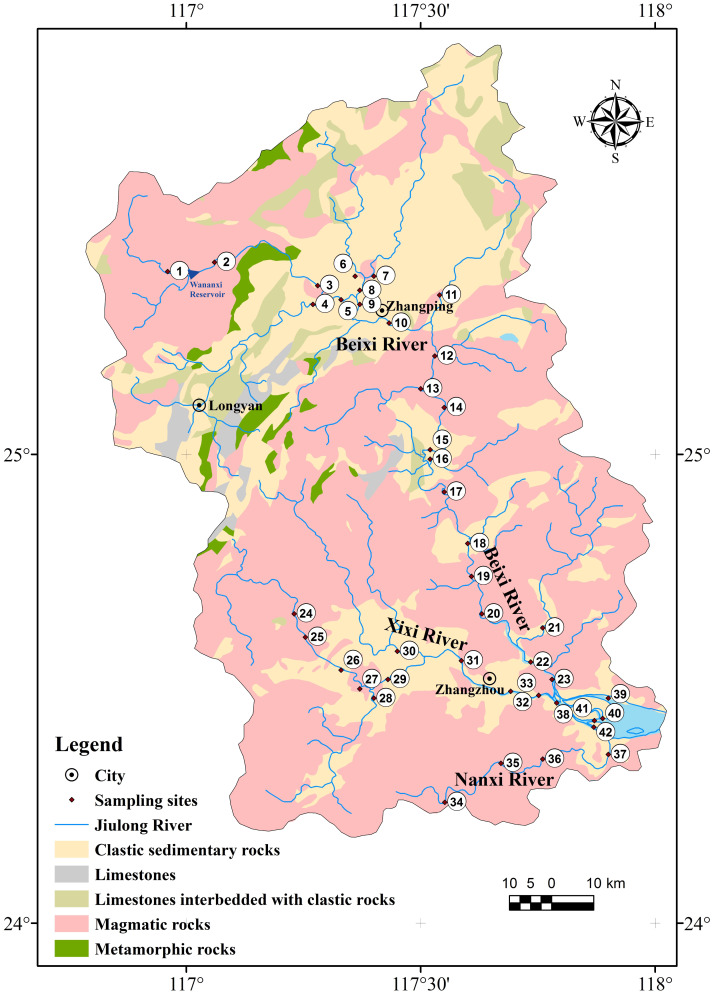
Lithologic distribution and the location of sampling sites in the Jiulongjiang River basin.

A total of 42 river water samples were collected in the high-flow season (July) of 2014 (Fig. 1 and Table 1). The river water samples of 1–21, 24–33, and 34–37 were collected along with the Beixi River, Xixi River, and Nanxi River, respectively. The sites of 22, 23, and 38–42 were located in the estuary region. The physicochemical parameters of pH values and total dissolved solids (TDS) concentrations in river and estuary water were measured using a water quality monitor (YSI multi-parameter probe) on site. The HCO_3_^−^ concentrations were determined using titration with diluent HCl solution (Liu & Han, 2021). All river water samples were hoisted by a clean plastic bucket from the center of the river on bridges and ferries. The samples were collected at a depth about 0.5 m below the water surface. Water samples were stored in a 50L LDPE bag, which had been pre-cleaned with 10% nitric acid. The collected river waters were filtered through a 0.22 µm cellulose acetate membrane (Millipore) to separate SPM samples within 24 h. Compared to the 0.45 µm size, the 0.22 µm size was more widely employed to distinguish SPM and dissolved solutes in natural river water (Zeng & Han, 2020; Zeng, Han & Yang, 2020). An important reason is that the 0.22 µm filter can separate most particles or colloids associated with anthropogenic sources, such as living substances and biopolymer aggregates (Jackson & Burd, 2015). The SPM samples absorbed on the surface of the filter membrane were collected through washing by deionized water and then dried on a hotplate at 55 °C.

**Table 1:** Location and the pH, TDS, and HCO_3_^−^ concentration of river water at different sampling sites in the Jiulongjiang River. (T) indicates the site located in tributary of the Beixi River.

Sampling site	Longitude	Latitude	Altitude	pH	TDS (mg/L)	HCO_3_^−^ (mg/L)
Beixi River						
1	116.96°E	25.39°N	384 m	7.58	27.1	7.50
2	117.06°E	25.41°N	282 m	6.94	32.8	7.87
3	117.28°E	25.36°N	206 m	6.72	43.4	11.5
4(T)	117.27°E	25.32°N	197 m	7.03	229	32.9
5	117.33°E	25.33°N	173 m	6.96	118	21.1
6(T)	117.36°E	25.38°N	182 m	7.30	54.7	22.5
7(T)	117.40°E	25.38°N	174 m	7.91	93.4	39.4
8(T)	117.37°E	25.35°N	162 m	7.65	86.9	36.6
9	117.37°E	25.32°N	172 m	7.10	96.1	26.0
10	117.44°E	25.28°N	159 m	7.13	121	23.8
11(T)	117.54°E	25.34°N	177 m	7.53	76.1	38.1
12	117.53°E	25.21°N	143 m	7.09	99.7	22.5
13	117.5°E	25.14°N	153 m	7.35	43.6	11.9
14	117.55°E	25.10°N	109 m	6.90	90.9	17.9
15	117.52°E	25.01°N	89 m	7.31	95.4	21.8
16	117.52°E	24.99°N	83 m	7.60	59.8	15.0
17	117.55°E	24.92°N	35 m	7.20	91.2	20.1
18	117.60°E	24.81°N	27 m	7.16	87.3	19.2
19	117.60°E	24.74°N	16 m	7.15	87.0	17.9
20	117.63°E	24.66°N	8 m	7.07	88.1	17.9
21(T)	117.76°E	24.63°N	13 m	7.58	77.5	20.7
Xixi River						
24	117.23°E	24.66°N	136 m	7.18	52.1	15.0
25	117.25°E	24.61°N	78 m	7.31	55.8	14.8
26	117.33°E	24.54°N	37 m	7.38	59.3	15.6
27	117.37°E	24.50°N	23 m	7.40	59.9	15.0
28	117.40°E	24.48°N	24 m	6.71	156	9.3
29	117.43°E	24.52°N	20 m	7.13	91.2	14.6
30	117.45°E	24.58°N	27 m	6.87	82.7	26.5
31	117.59°E	24.56°N	16 m	7.04	106	21.6
32	117.69°E	24.50°N	1 m	6.94	141	37.5
33	117.75°E	24.49°N	1 m	7.01	173	49.0
Nanxi River						
34	117.55°E	24.26°N	70 m	7.29	70.4	15.2
35	117.67°E	24.34°N	16 m	7.00	91.0	20.5
36	117.76°E	24.35°N	7 m	6.96	120	26.2
37	117.90°E	24.36°N	−1 m	6.99	299	38.43
Estuary						
22	117.74°E	24.56°N	7 m	7.00	90.3	20.7
23	117.78°E	24.52°N	29 m	7.08	88.8	19.6
38	117.79°E	24.47°N	7 m	6.95	99.5	22.5
39	117.90°E	24.48°N	−2 m	7.05	105	20.7
40	117.89°E	24.44°N	0 m	7.02	720	32.0
41	117.87°E	24.43°N	0 m	6.99	97.2	20.5
42	117.86°E	24.42°N	5 m	7.00	99.0	21.6

### Analytical methods

The SPM samples were digested to dissolved loads according to the modified method by Li & Han (2021). The procedure was showed in detail, dried SPM sample was weighed 100 mg, put into a cleaned PFA digestion tank digested, added one mL pure HF and three mL pure HNO_3_, heated keeping at 140 °C for 3 days (Li et al., 2020). Finally, the solid sample was transformed into a solution, and then stored in a 2% HNO_3_ solution for analyzing the concentrations of REEs and major elements. For the entire procedure, the blank samples and standard samples (GBW07404 and GBW07120) were treated the same as the samples, to monitor procedural reliability. The recovery rate of Ce was more than 90% and over 95% for other REEs during the digestion procedure. The concentrations of REEs (including La, Ce, Pr, Nd, Sm, Eu, Gd, Tb, Dy, Ho, Er, Tm, Yb, and Lu) in SPM were determined by an inductively coupled plasma mass spectrometry (ICP-MS, ELAN DRC-e, Perkin Elmer, Waltham, Massachusetts, USA), and the concentrations of major elements (including Al, Ca, Fe, K, Mg, Mn, Na, and Ti) were analyzed by an inductively coupled plasma optical emission spectroscopy (ICP-OES, Optima 5300DV, PerkinElmer, Waltham, Massachusetts, USA) (Liu, Han & Li, 2021). To restrict the mass interferences of BaO^+^ on Eu during the instrument testing process, a liquid-liquid extraction method was employed to remove Ba efficiently, as well as other matrix elements (Shabani & Masuda, 1991). The precision and accuracy of measurement were evaluated by repeated analysis of standard solution with multi-element, which were better than ±3% for major elements and better than ±5% for REEs, respectively.

### Chemical index of alteration

The chemical index of alteration (CIA) of SPM can indicate the degree of weathering (*i.e.,* the evolution degree from luminum–silicate minerals (especially feldspar) to clay minerals) in the source region (Nesbitt & Young, 1982). The higher CIA value means the more intensive leaching of Na, K, and Ca in silicate minerals, that is the stronger chemical weathering. The CIA > 80% indicates strong chemical weathering; 60% < CIA < 80% indicates moderate chemical weathering; CIA < 60% indicates weak chemical weathering. The CIA can be obtained by the calculation of molecular proportions, as the formula:

(1) \begin{eqnarray*}\mathrm{CIA}=[{\mathrm{Al}}_{2}{\mathrm{O}}_{3}/{\mathrm{Al}}_{2}{\mathrm{O}}_{3}+{\mathrm{CaO}}^{\ast }+{\mathrm{Na}}_{2}\mathrm{O}+{\mathrm{K}}_{2}\mathrm{O}\times 100]\end{eqnarray*}

where CaO* (mol/kg) is the molecular mass concentration of CaO incorporated in the silicate fraction of the rock. A correction is made for carbonate and apatite content (McLennan, 1993). In brief, if CaO/Na_2_O (mole ratio) > 1, the mole fraction of CaO* is replaced by the mole fraction of Na_2_O; if CaO/Na_2_O < 1, CIA is calculated directly using the mole fraction of CaO.

### REE fractionation indexes and anomalies

The Post Archean Australia Shale (PAAS)-normalized REE patterns for SPM was calculated as the formula (Han et al., 2009):

(2) \begin{eqnarray*}{\mathrm{REE}}_{\mathrm{N}}={\mathrm{REE}}_{\mathrm{SPM}}/{\mathrm{REE}}_{\mathrm{PAAS}}\end{eqnarray*}

The PAAS-normalized La/Yb ratio ((La/Yb)_N_), La/Sm ratio ((La/Sm)_N_), and Sm/Yb ratio ((Sm/Yb)_N_) indicate the relative fractionation between LREE and HREE, between LREE and MREE, and between MREE and HREE in SPM, respectively (Xu & Han, 2009):

(3) \begin{eqnarray*}(\mathrm{La}/\mathrm{Y b})_{\mathrm{N}}=(\mathrm{La}/\mathrm{Y b})_{\mathrm{SPM}}/(\mathrm{La}/\mathrm{Y b})_{\mathrm{PAAS}}\end{eqnarray*}

(4) \begin{eqnarray*}(\mathrm{La}/\mathrm{Sm})_{\mathrm{N}}=(\mathrm{La}/\mathrm{Sm})_{\mathrm{SPM}}/(\mathrm{La/Sm})_{\mathrm{PAAS}}\end{eqnarray*}

(5) \begin{eqnarray*}(\mathrm{Sm/Y b})_{\mathrm{N}}=(\mathrm{Sm/Y b})_{\mathrm{SPM}}/(\mathrm{Sm/Y b})_{\mathrm{PAAS}}\end{eqnarray*}

The anomalies of Ce (δCe), Eu (δEu), and Gd (δGd) are calculated as the formulas (Hissler et al., 2015; Olivarez & Owen, 1991):

(6) \begin{eqnarray*}\mathrm{\delta }\mathrm{Ce}={\mathrm{Ce}}_{\mathrm{SPM}}/{\mathrm{Ce}}_{\mathrm{PAAS}}/(0.5\times {\mathrm{La}}_{\mathrm{SPM}}/{\mathrm{La}}_{\mathrm{PAAS}}+0.5\times {\mathrm{Pr}}_{\mathrm{SPM}}/{\mathrm{Pr}}_{\mathrm{PAAS}})\end{eqnarray*}

(7) \begin{eqnarray*}\mathrm{\delta }\mathrm{Eu}={\mathrm{Eu}}_{\mathrm{SPM}}/{\mathrm{Eu}}_{\mathrm{PAAS}}/(0.67\times {\mathrm{Sm}}_{\mathrm{SPM}}/{\mathrm{Sm}}_{\mathrm{PAAS}}+0.33\times {\mathrm{Tb}}_{\mathrm{SPM}}/{\mathrm{Tb}}_{\mathrm{PAAS}})\end{eqnarray*}

(8) \begin{eqnarray*}\mathrm{\delta }\mathrm{Gd}={\mathrm{Gd}}_{\mathrm{SPM}}/{\mathrm{Gd}}_{\mathrm{PAAS}}/(0.33\times {\mathrm{Sm}}_{\mathrm{SPM}}/{\mathrm{Sm}}_{\mathrm{PAAS}}+0.67\times {\mathrm{Tb}}_{\mathrm{SPM}}/{\mathrm{Tb}}_{\mathrm{PAAS}})\end{eqnarray*}

The positive and negative Ce and Eu anomalies are determined by the values of >1 and <1, respectively. The δGd > 1.6 implies the presence of anthropogenic Gd sources (Louis et al., 2020).

### Statistical analysis

Boxplot was used to show the ranges of the concentrations of ∑REE, ∑LREE, ∑MREE, and ∑HREE of SPM in different regions of the Jiulongjiang River basin. The normal distribution of the sample data set were tested *via* the Shapiro–Wilk test before Pearson correlation analysis. Pearson correlation coefficient determined the relationship between physicochemical parameters and REE fractionation proxies of SPM. All statistical analyses were performed by the SPSS 18.0 software (SPSS Inc., Chicago, IL, USA) and all graphs were drawn by SigmaPlot 12.5 software (Systat Software GmbH, Erkrath, Germany).

## Results

### Hydrochemical characteristics

The pH, TDS, and HCO_3_^−^ concentration of river water at different sampling sites in the Jiulongjiang River are showed in Table 1. The pH values ranged from 6.7 to 7.9 (mean 7.2 ± 0.3), indicating the neutral and slightly alkaline river water. The TDS varied intensively with a range of 27.1–720 mg/L (mean 111 ± 107 mg/L). Moreover, the higher TDS mainly occurred in the water of the estuary region. The HCO_3_^−^ concentrations ranged from 7.5 mg/L to 49.0 mg/L (mean 22.1 ± 9.1 mg/L) and showed an increasing trend downstream. Particularly, the water of tributary in the Beixi River had higher HCO_3_^−^ concentrations compared to the main stream.

### The concentrations of major elements in SPM and CIA values

The concentrations of the major elements in SPM are shown in Table 2. The average Al, Ca, Fe, K, Mg, Mn, Na, and Ti concentrations in SPM were 151 ± 22.6 g/kg, 4.8 ± 7.8 g/kg, 49.6 ± 20.2 g/kg, 17.3 ± 3.7 g/kg, 4.3 ± 2.3 g/kg, 1.2 ± 0.5 g/kg, 2.1 ± 1.9 g/kg, and 2.9 ± 0.6 g/kg, respectively. Generally, the concentrations of the major elements slightly varied among the sites in the river, except for several sampling sites. For example, the No. 21 and 34 sites had relatively high Ca, Mg, and Na concentrations. However, Al and Fe concentrations at the 34 site were significantly lower than those at other sites, but not at the 21 site. Furthermore, most metal elements, besides Mn, were at a relatively high concentration at the 37 site. The CIA values varied among the sites in the river with a range of 83.2%–95.7% (89.6 ± 4.8%), except for the exceptionally low value of 63.7% at the 34 site (Table 3). Particularly, the CIA values in the tributary of the Beixi River were slightly lower than those in the main stream.

**Table 2:** The concentrations of major elements of SPM in the Jiulongjiang River. (T) indicates the site located in tributary of the Beixi River.

Sampling site	Al (g/kg)	Ca (g/kg)	Fe (g/kg)	K (g/kg)	Mg (g/kg)	Mn (g/kg)	Na (g/kg)	Ti (g/kg)
Beixi River								
1	175	2.83	45.9	17.4	3.45	2.92	1.84	2.33
2	167	2.59	38.7	12.8	2.89	0.86	1.24	1.79
3	171	2.49	45.5	15.3	3.14	0.97	1.18	2.08
4(T)	86.9	4.80	169	14.7	3.97	1.14	2.58	2.38
5	151	2.87	56.9	17.0	3.85	0.99	1.39	2.18
6(T)	153	3.76	51.6	22.6	5.40	0.76	1.73	3.07
7(T)	138	4.64	52.6	22.4	4.69	1.68	1.21	3.47
8(T)	139	4.72	51.3	22.6	4.84	1.61	1.25	3.36
9	141	3.68	54.6	19.9	5.01	0.97	1.70	2.91
10	153	2.83	53.9	18.2	3.94	1.09	1.24	2.56
11(T)	141	4.61	53.3	20.5	3.95	1.63	0.81	3.37
12	149	2.31	54.5	18.7	3.73	0.99	1.05	2.86
13	181	1.93	36.3	10.5	1.87	0.94	0.37	2.12
14	148	2.32	70.5	16.8	3.31	0.79	0.93	3.41
15	146	2.87	48.8	18.0	3.58	1.31	1.49	2.83
16	162	4.31	40.6	15.0	3.00	1.08	1.87	2.96
17	155	1.76	54.7	17.1	3.20	1.22	1.01	3.01
18	153	2.97	54.8	16.5	3.47	1.11	1.05	3.23
19	151	3.08	53.0	16.9	3.47	1.20	1.49	3.21
20	154	2.45	52.4	17.8	3.31	0.99	1.10	3.15
21(T)	152	12.9	47.4	19.2	8.04	1.43	7.18	4.04
Xixi River								
24	160	3.10	41.9	15.1	2.82	0.76	1.14	2.40
25	164	2.79	40.8	14.8	2.79	0.80	1.19	2.45
26	165	2.65	43.8	13.4	2.74	1.03	0.94	2.54
27	171	3.18	47.9	14.7	3.01	1.20	0.94	2.68
28	146	2.97	43.4	18.4	2.91	1.62	1.60	2.97
29	158	2.60	45.0	14.6	2.88	1.26	0.99	2.61
30	162	3.97	36.7	15.6	3.23	1.80	2.36	2.90
31	165	2.01	45.8	16.3	2.91	0.96	1.33	3.05
32	151	3.73	44.7	13.9	3.01	1.03	1.85	3.29
33	153	3.47	41.9	15.6	3.58	0.77	2.37	1.52
Nanxi River								
34	66.2	53.3	26.3	18.6	12.40	0.58	10.48	3.36
35	145	3.15	36.3	12.4	3.07	1.36	2.62	2.60
36	145	3.20	33.8	11.7	2.79	3.04	1.91	1.88
37	222	8.53	63.4	32.3	13.9	1.71	7.72	5.18
Estuary								
22	140	4.36	46.3	18.3	4.33	1.06	2.26	3.41
23	149	5.12	47.5	17.6	4.62	1.31	2.19	2.95
38	145	3.36	44.3	15.6	4.06	1.76	1.64	3.10
39	133	4.42	44.8	18.4	5.63	1.32	2.76	3.58
40	138	2.51	37.9	22.4	7.75	0.67	3.90	3.01
41	140	3.63	44.5	18.1	5.13	1.33	2.32	3.37
42	140	3.47	42.4	18.4	5.10	1.28	2.19	3.52

**Table 3:** The molality of metal oxides, CaO/Na_2_O ratio, and the CIA value of SPM in the Jiulongjiang River. (T) indicates the site located in tributary of the Beixi River.

Sampling site	Al_2_O_3_ (mol/kg)	CaO (mol/kg)	K_2_O (mol/kg)	Na_2_O (mol/kg)	CaO/Na_2_O	CIA (%)
Beixi River						
1	3.25	0.07	0.22	0.04	1.8	91.5
2	3.10	0.06	0.16	0.03	2.4	93.4
3	3.17	0.06	0.20	0.03	2.4	92.4
4(T)	1.61	0.12	0.19	0.06	2.1	83.9
5	2.80	0.07	0.22	0.03	2.4	90.9
6(T)	2.83	0.09	0.29	0.04	2.5	88.4
7(T)	2.56	0.12	0.29	0.03	4.4	88.0
8(T)	2.57	0.12	0.29	0.03	4.3	88.0
9	2.61	0.09	0.25	0.04	2.5	88.8
10	2.83	0.07	0.23	0.03	2.6	90.7
11(T)	2.61	0.12	0.26	0.02	6.5	89.7
12	2.77	0.06	0.24	0.02	2.5	90.8
13	3.36	0.05	0.13	0.01	6.0	95.7
14	2.74	0.06	0.21	0.02	2.9	91.6
15	2.70	0.07	0.23	0.03	2.2	90.3
16	3.00	0.11	0.19	0.04	2.6	91.7
17	2.87	0.04	0.22	0.02	2.0	91.7
18	2.83	0.07	0.21	0.02	3.2	91.9
19	2.80	0.08	0.22	0.03	2.4	90.9
20	2.86	0.06	0.23	0.02	2.6	91.4
21(T)	2.82	0.32	0.25	0.16	2.1	83.2
Xixi River						
24	2.96	0.08	0.19	0.02	3.1	92.8
25	3.04	0.07	0.19	0.03	2.7	92.4
26	3.06	0.07	0.17	0.02	3.2	93.6
27	3.17	0.08	0.19	0.02	3.9	93.2
28	2.71	0.07	0.24	0.03	2.1	90.0
29	2.93	0.06	0.19	0.02	3.0	92.7
30	3.00	0.10	0.20	0.05	1.9	90.9
31	3.07	0.05	0.21	0.03	1.7	91.9
32	2.80	0.09	0.18	0.04	2.3	91.5
33	2.83	0.09	0.20	0.05	1.7	90.4
Nanxi River						
34	1.23	1.33	0.24	0.23	5.8	63.7
35	2.69	0.08	0.16	0.06	1.4	90.6
36	2.68	0.08	0.15	0.04	1.9	92.1
37	4.11	0.21	0.41	0.17	1.3	84.6
Estuary						
22	2.60	0.11	0.23	0.05	2.2	88.7
23	2.75	0.13	0.23	0.05	2.7	89.3
38	2.69	0.08	0.20	0.04	2.3	90.6
39	2.46	0.11	0.24	0.06	1.8	87.2
40	2.56	0.06	0.29	0.08	0.7	85.6
41	2.60	0.09	0.23	0.05	1.8	88.7
42	2.60	0.09	0.24	0.05	1.8	88.4

**Table 4:** The concentration of REEs of SPM in the Jiulongjiang River. (T) indicates the site located in tributary of the Beixi River.

Sampling site	La (mg/kg)	Ce (mg/kg)	Pr (mg/kg)	Nd (mg/kg)	Sm (mg/kg)	Eu (mg/kg)	Gd (mg/kg)	Tb (mg/kg)	Dy (mg/kg)	Ho (mg/kg)	Er (mg/kg)	Tm (mg/kg)	Yb (mg/kg)	Lu (mg/kg)
Beixi River														
1	253	332	46.6	162	31.5	4.45	37.3	3.90	19.7	3.46	10.1	1.28	8.19	1.11
2	148	237	27.1	100	17.3	2.44	21.3	2.07	10.5	1.91	5.78	0.77	5.12	0.71
3	131	221	24.2	82.3	16.1	2.08	20.6	2.03	10.4	1.88	5.54	0.72	4.83	0.66
4(T)	52.2	86.2	10.5	42.4	10.3	2.27	15.2	1.73	8.61	1.56	4.26	0.53	3.38	0.49
5	92.1	162	17.9	63.3	12.5	1.76	15.8	1.59	8.15	1.49	4.40	0.59	3.94	0.55
6(T)	47.5	82.5	9.7	36.5	8.01	1.41	9.76	1.12	6.06	1.16	3.48	0.49	3.30	0.48
7(T)	62.6	108	12.3	45.8	9.20	1.78	11.7	1.18	6.03	1.13	3.36	0.44	2.91	0.43
8(T)	64.2	110	12.5	46.8	9.42	1.80	11.8	1.20	6.22	1.17	3.48	0.46	3.10	0.44
9	86.7	152	16.8	61.5	12.7	2.23	16.3	1.65	8.36	1.51	4.38	0.57	3.73	0.53
10	70.1	127	13.8	48.2	9.89	1.49	12.4	1.28	6.56	1.22	3.64	0.49	3.32	0.47
11(T)	55.9	98.2	10.8	39.1	7.63	1.42	9.29	0.91	4.62	0.86	2.58	0.34	2.31	0.33
12	87.3	164	17.4	61.2	12.3	2.01	15.2	1.54	7.96	1.49	4.43	0.59	3.87	0.55
13	95.5	188	20.7	72.7	15.6	2.15	18.5	1.95	10.5	2.05	6.44	0.93	6.42	0.93
14	91.4	168	18.4	66.3	13.2	2.08	16.5	1.66	8.64	1.60	4.84	0.66	4.37	0.62
15	84.1	151	16.0	58.7	12.0	1.87	14.9	1.57	8.12	1.55	4.70	0.64	4.21	0.61
16	101	176	20.1	69.9	14.3	2.45	17.6	1.80	9.48	1.83	5.62	0.78	5.33	0.78
17	79.8	156	16.1	56.9	11.7	1.90	14.5	1.44	7.51	1.42	4.27	0.59	3.91	0.56
18	77.2	152	16.0	57.5	11.7	1.95	14.6	1.48	7.62	1.44	4.38	0.60	3.98	0.56
19	77.2	146	15.5	55.8	11.7	1.87	14.8	1.52	8.07	1.54	4.68	0.64	4.26	0.62
20	74.1	146	15.3	53.6	11.3	1.77	14.0	1.41	7.35	1.40	4.26	0.58	3.91	0.56
21(T)	76.3	140	15.1	53.8	10.8	2.29	12.9	1.26	6.58	1.26	3.86	0.51	3.46	0.49
Xixi River														
24	81.4	144	16.1	56.3	11.7	1.76	14.4	1.53	8.14	1.54	4.59	0.63	4.21	0.59
25	88.1	150	16.5	58.9	12.1	1.81	15.1	1.61	8.58	1.61	4.82	0.67	4.48	0.65
26	86.0	154	16.9	59.9	12.2	1.85	15.1	1.57	8.43	1.59	4.83	0.66	4.47	0.64
27	86.9	164	17.9	64.2	12.9	1.97	15.9	1.65	8.73	1.63	4.92	0.67	4.48	0.64
28	410	334	75.7	274	49.2	6.65	57.9	6.86	34.9	6.53	18.4	2.30	14.3	1.97
29	140	183	25.3	89.7	17.3	2.60	21.0	2.28	11.8	2.21	6.44	0.83	5.44	0.76
30	106	170	19.3	69.2	13.3	2.16	16.8	1.71	9.11	1.74	5.49	0.77	5.39	0.8
31	113	186	21.5	76.7	15.0	2.33	18.7	1.87	9.79	1.84	5.51	0.73	4.89	0.69
32	90.9	161	17.8	62.3	12.1	2.01	15.1	1.48	7.59	1.41	4.26	0.58	3.85	0.55
33	87.9	150	16.3	60.0	11.6	1.89	14.5	1.44	7.43	1.39	4.18	0.56	3.80	0.55
Nanxi River														
34	25.3	45.1	5.1	20.0	4.08	0.91	4.48	0.43	2.18	0.39	1.18	0.16	1.07	0.15
35	106	157	18.2	63.8	12.3	1.93	15.4	1.59	8.71	1.68	5.26	0.73	4.91	0.72
36	81.6	120	15.5	53.0	10.5	1.83	12.4	1.34	7.33	1.43	4.45	0.63	4.30	0.62
37	86.5	145	16.3	64.6	12.4	2.14	16.9	1.58	8.40	1.59	4.95	0.70	4.37	0.66
Estuary														
22	63.7	121	12.9	46.6	9.56	1.71	11.8	1.20	6.26	1.18	3.61	0.50	3.29	0.47
23	50.5	92.4	10.4	39.2	7.72	1.38	9.65	0.98	5.18	1.00	3.03	0.42	2.83	0.41
38	63.9	119	13.1	47.2	9.69	1.61	11.5	1.20	6.34	1.21	3.65	0.48	3.25	0.45
39	50.5	91.6	9.7	35.2	7.59	1.33	9.36	0.98	5.23	0.99	3.00	0.41	2.73	0.40
40	39.3	73.3	7.8	29.0	5.66	1.06	6.91	0.66	3.44	0.66	1.97	0.27	1.81	0.26
41	58.9	106	11.5	42.5	8.87	1.49	10.9	1.13	6.00	1.15	3.46	0.47	3.12	0.45
42	58.8	108	11.7	43.4	8.82	1.52	11.0	1.11	5.93	1.13	3.43	0.47	3.12	0.45

### REE concentrations of SPM

**Figure 2:**
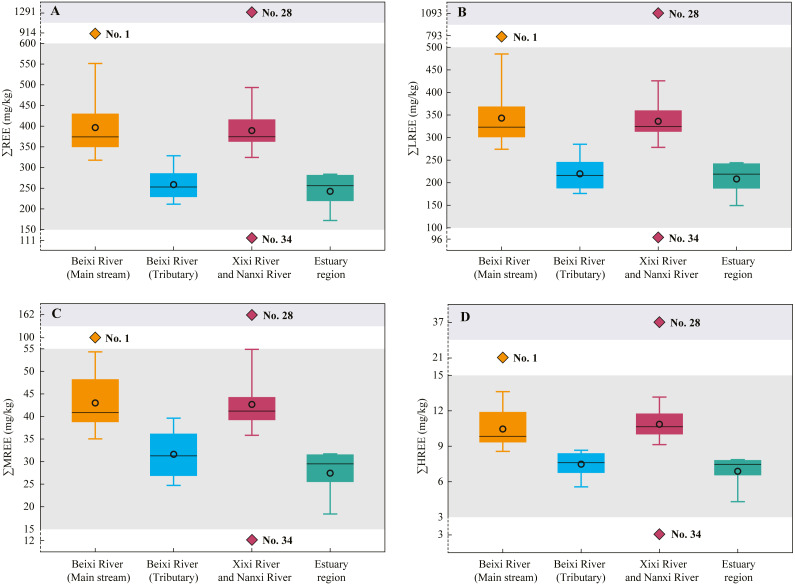
The ∑REE (A), ∑LREE (B), ∑MREE (C), and ∑HREE (D) concentrations of SPM in different regions of the Jiulongjiang River basin.

The concentrations of single REE in SPM at different sampling sites are shown in Table 4. The total REE (∑REE) concentrations of SPM ranged from 111 mg/kg to 1,292 mg/kg with an average of 376 ± 191 mg/kg (Fig. 2), which were higher than those of the UCC (146 mg/kg) (Taylor & McLennan, 1985), the local soils of Fujian Province (198 mg kg^−1^) (Chen et al., 1992), the SPM of world rivers (175 mg/kg) (Viers, Dupre & Gaillardet, 2009), and the PAAS (185 mg/kg) (Taylor & McLennan, 1985)). The LREEs accounted for the largest proportion of total REEs in SPM at all sites. The ∑LREE concentrations of SPM ranged from 95.5 mg/kg to 1093 mg/kg with an average of 324 ± 164 mg/kg. The ∑MREE and ∑HREE concentrations of SPM varied from 12.5 mg/kg to 162 mg/kg (mean 42.2 ± 22.9 mg/kg) and from 2.6 mg/kg to 36.9 mg/kg (mean 10.2 ± 5.1 mg/kg), respectively. For spatial distribution, the ∑REE ∑LREE, ∑MREE and ∑HREE concentrations of SPM in the main stream of Beixi River were similar to those in the Xixi River and Nanxi River (Fig. 2). However, these concentrations in the tributary of Beixi River were significantly lower than those in the main stream, but near to those in the estuary region.

### PAAS-normalized REE patterns of SPM

**Figure 3:**
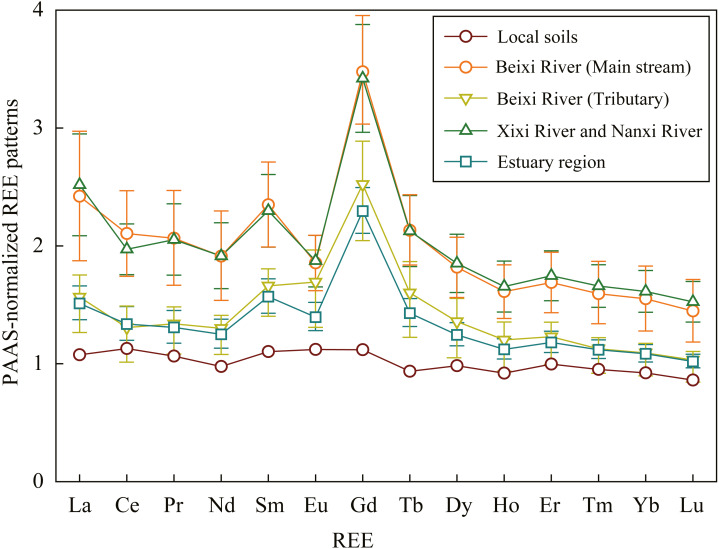
The PAAS-normalized REE patterns for average SPM and local soils. The data of local soils of Fujian Province were cited from Chen et al. (1992).

**Figure 4:**
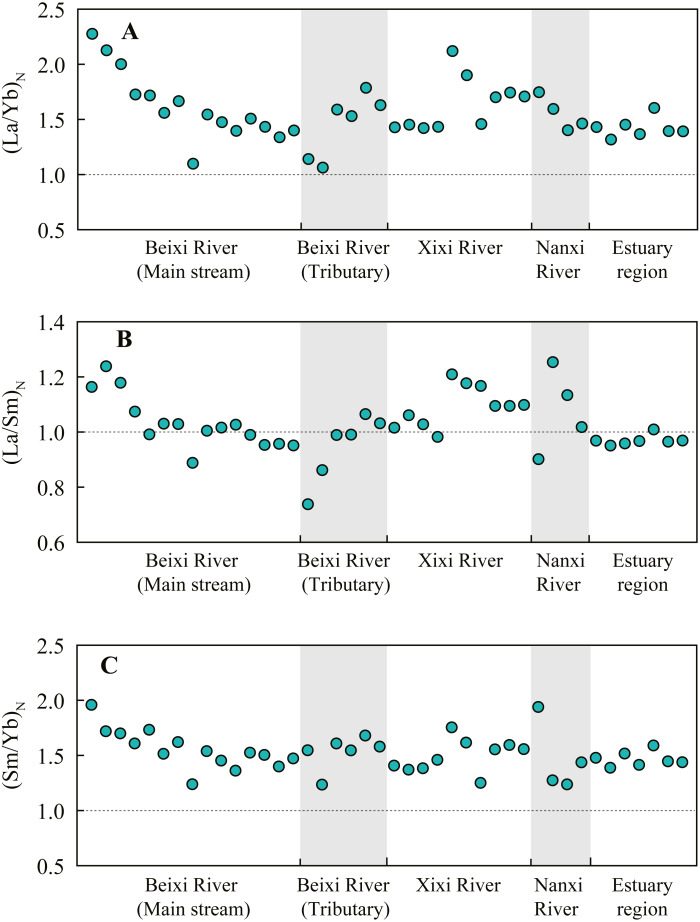
The (La/Yb)_N_ ratio (A), (La/Sm)_N_ ratio (B), and (Sm/Yb)_N_ ratio (C) of SPM at the sampling sites of different regions in the Jiulongjiang River basin.

The PAAS-normalized REE patterns of SPM at each site were showed in Table 5. The PAAS-normalized REE ratios of SPM at most sites were larger than 1, except for the parts of REEs at the 11, 34, and 40 sites. The pattern of average SPM in the main stream of Beixi River was almost identical to that in the Xixi River and Nanxi River (Fig. 3). Additionally, the pattern in the tributary of Beixi River was similar to that in the estuary region. Overall, the enrichment of REEs in SPM was larger in the main stream than that in tributary and estuary. The (La/Yb)_N_ ratio, (La/Sm)_N_ ratio, and (Sm/Yb)_N_ ratio of SPM at different sites are shown in Fig. 4. The (La/Yb)_N_ and (Sm/Yb)_N_ ratios at all sites were higher than 1, indicating the enrichment of LREE and MREE relative to HREE in SPM. The (La/Yb)_N_ and (Sm/Yb)_N_ ratios generally showed a decreasing trend downstream in the Beixi River. The (La/Sm)_N_ ratio of SPM varied within a wide range of 0.7–1.3, indicating the strong spatial variability between LREE and MREE fractionation in the river.

### Ce, Eu, and Gd anomalies in SPM

**Figure 5:**
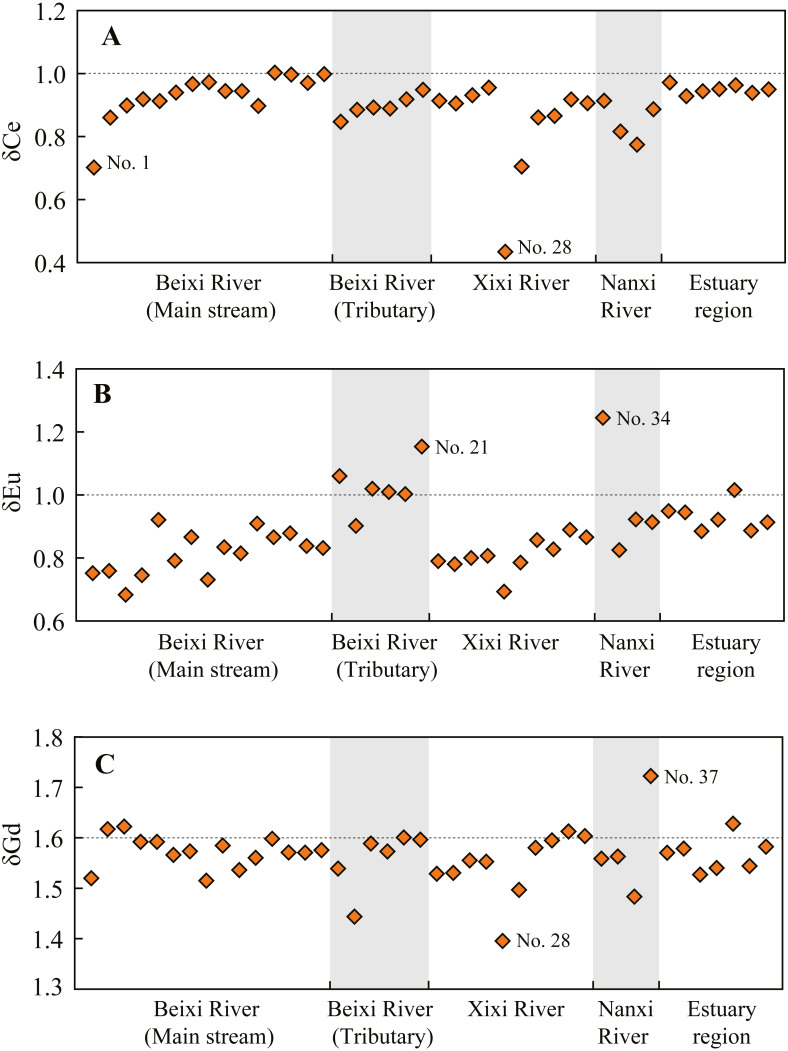
The Ce anomaly (A), Eu anomaly (B), and Gd anomaly (C) of SPM at the sampling sites of different regions in the Jiulongjiang River basin.

The Ce, Eu, and Gd anomalies in SPM at different sites are shown in Fig. 5. The Ce of SPM at most sites showed a slight negative anomaly (δCe: 0.8–1.0), except for the relatively large negative anomaly at the 1 and 28 sites with a δCe value of 0.70 and 0.43, respectively. The Eu of SPM at most sites showed negative anomaly (δEu: 0.7–1.0). Exceptionally, a positive Eu anomaly occurred at the 21 and 34 sites with a δEu value of 1.15 and 1.24, respectively. Additionally, the δEu value generally showed an increasing trend along with the flow direction of the main stream. The δGd values of SPM at most sites ranged from 1.5 to 1.6, except for a relatively higher δGd value at the 37 site (1.72) and a relatively lower value at the 28 site (1.40). This result indicates that the SPM in the Jiulongjiang River is almost not affected by anthropogenic Gd sources.

## Discussion

### Variation of REE concentrations and PAAS-normalized REE ratios in SPM

#### Controls of REE concentrations in SPM of river water by weathering processes

The clay minerals of SPM mainly derived from the weathering of rocks are important carriers of REE (Migaszewski & Galuszka, 2015). Thus, the REE concentrations of SPM are closely associated with the lithologic distribution in a basin. In the main stream of Beixi River, the PAAS-normalized REE ratios of SPM positively correlated with Al concentrations and CIA values, and negatively correlated with TDS, Fe, K, and Mg concentrations (Table 6). These results indicated that the REE concentrations of SPM were dominated by silicate weathering in the main stream, similar to the report from the downstream of Zhujiang River (Xu & Han, 2009). In the basin, the weathering of magmatic rocks and clastic sedimentary rocks contributes to abundant Al-silicate minerals (clay minerals) into soils. These clay minerals, which carry most REEs, are transferred into the river by soil erosion, resulting in the increment of REE concentration in SPM (Linders et al., 2018). Thus, the PAAS-normalized REE ratios positively correlated with Al concentrations in SPM.

In the tributary of Beixi River, the PAAS-normalized REE ratios of SPM positively correlated with Ca concentrations and negatively correlated with CIA values. These results indicated that the REE concentrations of SPM in the tributary were also affected by carbonate weathering. Han, Yang & Zeng (2021) reported a significant correlation relationship between Ca concentrations and REE concentrations of SPM upstream of the Zhujiang River mainly distributed by carbonate rocks. Moreover, the ∑REE, ∑LREE, ∑MREE, ∑HREE, and REE concentrations, PAAS-normalized REE ratios of SPM in the tributary were lower than those in the main stream (Figs. 2 and 3). A similar result about the low REE concentrations of SPM in the karst basin has been reported by Han et al. (2009). The CIA values of SPM in the tributary of the Beixi River were slightly lower than those in the main stream (Table 1). The result indicated that the SPM in the tributary consisted of many low-weathered carbonate minerals. However, the carbonate rock contains very few REEs, with a mean ∑REE concentration of 8.05 mg kg^−1^ (Han, Yang & Zeng, 2021). Thus, the river flowing through the carbonate rock region is characteristic of the low REE and high Ca concentrations in SPM (Xu & Han, 2009).

The ∑REE, ∑LREE, ∑MREE, and ∑HREE concentrations, PAAS-normalized REE ratios of SPM in the Xixi River and Nanxi River were near to those in the main stream of Beixi River (Figs. 2 and 3). Moreover, the correlation relationships (although not significantly) of the PAAS-normalized REE ratios with major element concentrations and CIA values were also similar in the three rivers (Table 6). These results indicated that the REE concentrations in the Xixi River and Nanxi River were also controlled by the weathering of magmatic rocks and clastic sedimentary rocks. However, the non-significant correlation relationships were likely attributed to the influences from human activities. Compared to the wide forest upstream of the Beixi River, the Xixi River and Nanxi River mainly pass through farmlands and residential areas (Liu & Han, 2020). Human activities can directly cause the increment of REE into the river and disturb REE fractionation by affecting physicochemical parameters of river water (Da Silva et al., 2018; Yang et al., 2021).

#### Influences of salinity on REE concentrations in SPM of estuary water

The PAAS-normalized REE ratios of SPM in the estuary region were near to those in the tributary of Beixi River (Fig. 3). The result is not attributed to the weathering of carbonate minerals like that in the tributary of the Beixi River. Theoretically, the characteristics of REE concentrations in the estuary region should inherit from the main stream of Beixi river, Xixi River, and Nanxi River because the three main rivers have the larger water discharge compared to the tributary of the Beixi River. In the estuary region, the PAAS-normalized REE ratios of SPM negatively correlated with K and Mg concentrations, and positively correlated with CIA values (Table 6), indicating the effects of silicate weathering on REE concentrations of SPM similar to the effects in the main stream of Beixi River. Compared to river water, the estuary water was lower in REE concentrations of SPM (Fig. 3), which was mainly attributed to REE removal. Elderfield, Upstill-Goddard & Sholkovitz (1990) found that significant REE removal (∼30%) occurred in the Connecticut, Delaware, Mullica, and Tamar estuaries. During the mixing of river water and seawater, the increment of salinity promotes the coagulation of colloidally associated Fe and Mn hydroxides (Migaszewski & Galuszka, 2015). The flocculations easily absorb REEs and transfer them into the sediments, resulting in significant removals of both dissolved and particulate REE. Thus, the coprecipitation of Fe and Mn hydroxides and REEs in the estuary explained the positive correlation (although non-significant) between the PAAS-normalized REE ratios and Fe (and Mn) concentrations of SPM (Table 6).

**Table 5:** The PAAS-normalized REE patterns for SPM in the Jiulongjiang River basin. (T) indicates the site located in tributary of the Beixi River.

Sampling site	La_N_	Ce_N_	Pr_N_	Nd_N_	Sm_N_	Eu_N_	Gd_N_	Tb_N_	Dy_N_	Ho_N_	Er_N_	Tm_N_	Yb_N_	Lu_N_
Beixi River														
1	6.61	4.17	5.28	4.77	5.68	4.12	8.01	5.06	4.21	3.49	3.53	3.12	2.90	2.58
2	3.86	2.98	3.07	2.96	3.12	2.26	4.58	2.69	2.24	1.93	2.03	1.88	1.82	1.65
3	3.43	2.77	2.74	2.43	2.91	1.93	4.42	2.64	2.21	1.90	1.94	1.76	1.71	1.53
4(T)	1.37	1.08	1.19	1.25	1.85	2.10	3.26	2.25	1.84	1.58	1.49	1.29	1.20	1.14
5	2.41	2.04	2.03	1.87	2.25	1.63	3.38	2.06	1.74	1.51	1.54	1.44	1.40	1.28
6(T)	1.24	1.04	1.10	1.08	1.44	1.31	2.09	1.45	1.29	1.17	1.22	1.20	1.17	1.12
7(T)	1.64	1.35	1.39	1.35	1.66	1.65	2.50	1.53	1.29	1.14	1.18	1.07	1.03	1.00
8(T)	1.68	1.38	1.42	1.38	1.70	1.67	2.52	1.56	1.33	1.18	1.22	1.12	1.10	1.02
9	2.27	1.90	1.90	1.81	2.29	2.06	3.49	2.14	1.79	1.53	1.54	1.39	1.32	1.23
10	1.84	1.60	1.56	1.42	1.78	1.38	2.67	1.66	1.40	1.23	1.28	1.20	1.18	1.09
11(T)	1.46	1.23	1.22	1.15	1.37	1.31	1.99	1.18	0.99	0.87	0.91	0.83	0.82	0.77
12	2.29	2.06	1.97	1.81	2.22	1.86	3.26	2.00	1.70	1.51	1.55	1.44	1.37	1.28
13	2.50	2.36	2.34	2.14	2.82	1.99	3.98	2.53	2.25	2.07	2.26	2.27	2.28	2.16
14	2.39	2.11	2.08	1.96	2.38	1.93	3.53	2.16	1.85	1.62	1.70	1.61	1.55	1.44
15	2.20	1.90	1.81	1.73	2.17	1.73	3.20	2.04	1.74	1.57	1.65	1.56	1.49	1.42
16	2.64	2.21	2.28	2.06	2.57	2.27	3.77	2.34	2.03	1.85	1.97	1.90	1.89	1.81
17	2.09	1.96	1.82	1.68	2.11	1.76	3.12	1.87	1.60	1.43	1.50	1.44	1.39	1.30
18	2.02	1.91	1.81	1.70	2.12	1.81	3.12	1.92	1.63	1.45	1.54	1.46	1.41	1.30
19	2.02	1.83	1.76	1.65	2.11	1.73	3.17	1.97	1.72	1.56	1.64	1.56	1.51	1.44
20	1.94	1.83	1.73	1.58	2.04	1.64	2.99	1.83	1.57	1.41	1.49	1.41	1.39	1.30
21(T)	2.00	1.76	1.71	1.59	1.94	2.12	2.77	1.64	1.41	1.27	1.35	1.24	1.23	1.14
Xixi River														
24	2.13	1.81	1.82	1.66	2.10	1.63	3.09	1.99	1.74	1.56	1.61	1.54	1.49	1.37
25	2.31	1.89	1.87	1.74	2.17	1.68	3.24	2.09	1.83	1.63	1.69	1.63	1.59	1.51
26	2.25	1.94	1.91	1.77	2.19	1.71	3.25	2.04	1.80	1.61	1.69	1.61	1.59	1.49
27	2.27	2.06	2.03	1.89	2.32	1.82	3.42	2.14	1.87	1.65	1.73	1.63	1.59	1.49
28	10.7	4.19	8.57	8.07	8.87	6.16	12.41	8.91	7.46	6.60	6.46	5.61	5.06	4.58
29	3.66	2.30	2.87	2.65	3.12	2.41	4.51	2.96	2.52	2.23	2.26	2.02	1.93	1.77
30	2.79	2.14	2.19	2.04	2.39	2.00	3.60	2.22	1.95	1.76	1.93	1.88	1.91	1.86
31	2.95	2.33	2.43	2.26	2.69	2.16	4.01	2.43	2.09	1.86	1.93	1.78	1.73	1.60
32	2.38	2.02	2.02	1.84	2.17	1.86	3.23	1.92	1.62	1.42	1.49	1.41	1.37	1.28
33	2.30	1.88	1.85	1.77	2.10	1.75	3.12	1.87	1.59	1.40	1.47	1.37	1.35	1.28
Nanxi River														
34	0.66	0.57	0.58	0.59	0.74	0.84	0.96	0.56	0.47	0.39	0.41	0.39	0.38	0.35
35	2.77	1.97	2.06	1.88	2.21	1.79	3.30	2.06	1.86	1.70	1.85	1.78	1.74	1.67
36	2.14	1.51	1.76	1.56	1.88	1.69	2.65	1.74	1.57	1.44	1.56	1.54	1.52	1.44
37	2.26	1.82	1.85	1.91	2.23	1.98	3.63	2.05	1.79	1.61	1.74	1.71	1.55	1.53
Estuary														
22	1.67	1.52	1.46	1.37	1.72	1.58	2.53	1.56	1.34	1.19	1.27	1.22	1.17	1.09
23	1.32	1.16	1.18	1.16	1.39	1.28	2.07	1.27	1.11	1.01	1.06	1.02	1.00	0.95
38	1.67	1.49	1.48	1.39	1.75	1.49	2.47	1.56	1.35	1.22	1.28	1.17	1.15	1.05
39	1.32	1.15	1.10	1.04	1.37	1.23	2.01	1.27	1.12	1.00	1.05	1.00	0.97	0.93
40	1.03	0.92	0.88	0.86	1.02	0.98	1.48	0.86	0.74	0.67	0.69	0.66	0.64	0.60
41	1.54	1.34	1.30	1.25	1.60	1.38	2.33	1.47	1.28	1.16	1.21	1.15	1.11	1.05
42	1.54	1.36	1.33	1.28	1.59	1.41	2.36	1.44	1.27	1.14	1.20	1.15	1.11	1.05

**Table 6:** Pearson correlation coefficient between physicochemical parameters and REEfractionation proxies of SPM in different regions of the Jiulongjiang River. The normal distribution of the sample data set were tested via the Shapiro-Wilk test before Pearson correlation analysis. Statistical significant correlation at the level of *P* < 0.05 (*) and *P* < 0.01 (**).

	Water pH	TDS	HCO_3_^−^	Al	Ca	Fe	K	Mg	Mn	Na	Ti	CIA
Beixi River (Main stream)												
La_N_	–0.38	–0.79^**^	–0.78^**^	0.58^*^	–0.02	–0.52	–0.58^*^	–0.35	–0.49	0.01	–0.71^**^	0.45
Ce_N_	–0.34	–0.86^**^	–0.85^**^	0.69^**^	–0.16	–0.55^*^	–0.70^**^	–0.51	–0.52	–0.17	–0.69^**^	0.61^*^
Pr_N_	–0.30	–0.85^**^	–0.84^**^	0.68^**^	–0.07	–0.57^*^	–0.70^**^	–0.48	–0.55^*^	–0.10	–0.71^**^	0.58^*^
Nd_N_	–0.30	–0.83^**^	–0.82^**^	0.62^*^	–0.06	–0.54^*^	–0.68^**^	–0.44	–0.54^*^	–0.08	–0.69^**^	0.55^*^
Sm_N_	–0.19	–0.90^**^	–0.85^**^	0.73^**^	–0.06	–0.62^*^	–0.77^**^	–0.55^*^	–0.53	–0.16	–0.66^*^	0.65^*^
Eu_N_	0.16	–0.75^**^	–0.57^*^	0.37	0.30	–0.47	–0.49	–0.29	–0.41	0.15	–0.21	0.33
Gd_N_	–0.26	–0.87^**^	–0.82^**^	0.67^**^	–0.02	–0.57^*^	–0.69^**^	–0.46	–0.53	–0.09	–0.66^*^	0.56^*^
Tb_N_	–0.17	–0.88^**^	–0.81^**^	0.70^**^	0.01	–0.61^*^	–0.73^**^	–0.50	–0.50	–0.11	–0.65^*^	0.60^*^
Dy_N_	–0.08	–0.90^**^	–0.82^**^	0.74^**^	–0.01	–0.64^*^	–0.79^**^	–0.58^*^	–0.47	–0.16	–0.61^*^	0.66^*^
Ho_N_	0.06	–0.93^**^	–0.83^**^	0.78^**^	–0.01	–0.69^**^	–0.84^**^	–0.67^**^	–0.40	–0.20	–0.55^*^	0.72^**^
Er_N_	0.15	–0.90^**^	–0.84^**^	0.81^**^	–0.04	–0.71^**^	–0.88^**^	–0.75^**^	–0.36	–0.26	–0.51	0.77^**^
Tm_N_	0.25	–0.86^**^	–0.81^**^	0.82^**^	–0.08	–0.70^**^	–0.91^**^	–0.81^**^	–0.31	–0.33	–0.45	0.82^**^
Yb_N_	0.28	–0.83^**^	–0.80^**^	0.84^**^	–0.07	–0.71^**^	–0.92^**^	–0.82^**^	–0.30	–0.34	–0.45	0.84^**^
Lu_N_	0.37	–0.80^**^	–0.75^**^	0.80^**^	–0.04	–0.70^**^	–0.89^**^	–0.82^**^	–0.24	–0.32	–0.38	0.81^**^
(La/Yb)_N_	–0.69^**^	–0.19	–0.21	–0.01	0.02	–0.02	0.09	0.30	–0.37	0.27	–0.52	–0.17
(La/Sm)_N_	–0.53	–0.37	–0.43	0.20	0.06	–0.24	–0.15	0.04	–0.29	0.28	–0.63^*^	0.03
(Sm/Yb)_N_	–0.74^**^	0.07	0.11	–0.30	0.01	0.25	0.38	0.58^*^	–0.36	0.27	–0.27	–0.41
δCe	0.22	0.39	0.36	–0.18	–0.44	0.34	0.23	–0.10	0.39	–0.47	0.60^*^	0.01
δEu	0.50	0.37	0.55^*^	–.63^*^	0.47	0.33	0.54^*^	0.46	0.28	0.43	0.77^**^	–0.55^*^
δGd	–0.71^**^	–0.09	–0.15	–0.11	–0.03	0.20	0.22	0.33	–0.27	0.27	–0.19	–0.24
Beixi River (Tributary)												
La_N_	0.58	–0.23	–0.14	0.34	0.83^*^	–0.38	0.07	0.71	0.60	0.69	0.80	–0.45
Ce_N_	0.56	–0.33	–0.25	0.44	0.88^*^	–0.46	0.10	0.79	0.54	0.73	0.86^*^	–0.43
Pr_N_	0.53	–0.22	–0.23	0.34	0.86^*^	–0.36	0.05	0.77	0.52	0.74	0.79	–0.51
Nd_N_	0.43	–0.01	–0.19	0.14	0.82^*^	–0.17	–0.10	0.70	0.48	0.74	0.63	–0.63
Sm_N_	–0.08	0.49	–0.27	–0.36	0.66	0.39	–0.53	0.51	0.10	0.73	0.11	–0.91^*^
Eu_N_	–0.21	0.62	–0.24	–0.49	0.64	0.53	–0.69	0.42	0.07	0.71	–0.01	–0.95^**^
Gd_N_	–0.38	0.83^*^	–0.08	–0.74	0.32	0.76	–0.77	0.11	–0.04	0.45	–0.35	–0.86^*^
Tb_N_	–0.60	0.90^*^	–0.12	–0.83^*^	0.09	0.88^*^	–0.79	–0.05	–0.31	0.28	–0.60	–0.77
Dy_N_	–0.60	0.85^*^	–0.21	–0.77	0.12	0.84^*^	–0.75	0.02	–0.38	0.33	–0.57	–0.79
Ho_N_	–0.59	0.79	–0.29	–0.71	0.17	0.79	–0.71	0.10	–0.42	0.38	–0.52	–0.81
Er_N_	–0.49	0.65	–0.43	–0.54	0.32	0.64	–0.60	0.30	–0.42	0.52	–0.34	–0.86^*^
Tm_N_	–0.46	0.44	–0.61	–0.33	0.32	0.46	–0.41	0.43	–0.58	0.54	–0.25	–0.78
Yb_N_	–0.38	0.30	–0.69	–0.17	0.42	0.32	–0.32	0.56	–0.56	0.62	–0.11	–0.77
Lu_N_	–0.38	0.32	–0.66	–0.20	0.34	0.35	–0.30	0.50	–0.60	0.56	–0.17	–0.73
(La/Yb)_N_	0.72	–0.40	0.41	0.39	0.35	–0.53	0.26	0.13	0.90^*^	0.08	0.71	0.23
(La/Sm)_N_	0.81	–0.73	0.18	0.73	0.36	–0.83^*^	0.58	0.32	0.73	0.10	0.875^*^	0.37
(Sm/Yb)_N_	0.40	0.22	0.61	–0.22	0.25	0.06	–0.27	–0.15	0.88^*^	0.05	0.27	–0.09
δCe	0.48	–0.69	–0.38	0.76	0.73	–0.72	0.31	0.72	0.34	0.57	0.92^**^	–0.04
δEu	0.11	0.32	–0.14	–0.19	0.82^*^	0.19	–0.53	0.53	0.44	0.77	0.38	–0.78
δGd	0.55	0.04	0.47	–0.01	0.43	–0.14	–0.12	0.10	0.92^**^	0.22	0.50	–0.14
Xixi River and Nanxi River												
La_N_	–0.20	–0.13	–0.24	–0.15	–0.27	–0.06	–0.12	–0.18	–0.08	–0.18	0.03	0.11
Ce_N_	0.04	–0.23	–0.24	0.00	–0.30	0.15	–0.06	–0.21	–0.48	–0.27	0.15	0.19
Pr_N_	–0.08	–0.18	–0.30	–0.11	–0.31	0.04	–0.12	–0.21	–0.14	–0.26	0.06	0.20
Nd_N_	–0.09	–0.01	–0.20	0.10	–0.13	0.23	0.10	0.00	–0.17	–0.07	0.22	0.02
Sm_N_	0.04	–0.12	–0.32	0.08	–0.21	0.22	0.04	–0.07	–0.24	–0.17	0.17	0.12
Eu_N_	–0.26	0.19	–0.05	0.20	0.04	0.31	0.23	0.15	0.01	0.09	0.35	–0.15
Gd_N_	0.00	0.05	–0.22	0.28	–0.01	0.39	0.26	0.14	–0.26	0.03	0.36	–0.08
Tb_N_	0.12	–0.19	–0.43	0.08	–0.23	0.17	0.02	–0.09	–0.22	–0.20	0.14	0.16
Dy_N_	0.13	–0.23	–0.50	0.07	–0.23	0.13	0.01	–0.09	–0.16	–0.20	0.13	0.17
Ho_N_	0.10	–0.23	–0.52	0.07	–0.22	0.10	0.01	–0.08	–0.10	–0.17	0.14	0.15
Er_N_	0.00	–0.18	–0.49	0.10	–0.14	0.07	0.05	–0.02	0.01	–0.08	0.19	0.06
Tm_N_	–0.06	–0.14	–0.49	0.17	–0.02	0.06	0.12	0.07	0.14	0.04	0.28	–0.04
Yb_N_	–0.07	–0.31	–0.52	–0.01	–0.18	–0.15	–0.07	–0.12	0.16	–0.12	0.11	0.11
Lu_N_	–0.15	–0.19	–0.42	0.09	–0.03	–0.11	0.04	0.00	0.23	0.03	0.19	–0.04
(La/Yb)_N_	–0.30	0.13	0.24	–0.26	–0.23	0.05	–0.12	–0.16	–0.33	–0.15	–0.06	0.03
(La/Sm)_N_	–0.60^*^	–0.08	0.05	–0.53	–0.23	–0.56	–0.36	–0.27	0.29	–0.06	–0.24	–0.01
(Sm/Yb)_N_	0.10	0.23	0.25	0.12	–0.06	0.48	0.15	0.04	–0.57	–0.09	0.12	0.02
δCe	0.41	–0.02	0.15	0.28	0.13	0.30	0.16	0.09	–0.53	0.02	0.14	–0.01
δEu	–0.74^**^	0.74^**^	0.78^**^	0.18	0.58^*^	0.14	0.36	0.47	0.65^*^	0.62^*^	0.30	–0.62^*^
δGd	–0.31	0.79^**^	0.63^*^	0.74^**^	0.79^**^	0.75^**^	0.82^**^	0.80^**^	–0.17	0.81^**^	0.78^**^	–0.83^**^
Estuary region												
La_N_	–0.62	–0.78^*^	–0.68	0.26	0.23	0.57	–0.80^*^	–0.88^**^	0.67	–0.88^**^	0.38	0.81^*^
Ce_N_	–0.63	–0.74	–0.63	0.26	0.21	0.55	–0.77^*^	–0.87^*^	0.62	–0.85^*^	0.36	0.79^*^
Pr_N_	–0.60	–0.77^*^	–0.66	0.37	0.25	0.59	–0.82^*^	–0.91^**^	0.68	–0.90^**^	0.27	0.86^*^
Nd_N_	–0.57	–0.78^*^	–0.68	0.43	0.27	0.61	–0.82^*^	–0.92^**^	0.68	–0.92^**^	0.24	0.88^**^
Sm_N_	–0.58	–0.82^*^	–0.72	0.29	0.28	0.62	–0.84^*^	–0.91^**^	0.72	–0.91^**^	0.36	0.85^*^
Eu_N_	–0.50	–0.80^*^	–0.72	0.30	0.35	0.65	–0.78^*^	–0.91^**^	0.60	–0.87^*^	0.36	0.80^*^
Gd_N_	–0.51	–0.85^*^	–0.76^*^	0.29	0.34	0.65	–0.82^*^	–0.92^**^	0.68	–0.91^**^	0.41	0.83^*^
Tb_N_	–0.51	–0.87^*^	–0.79^*^	0.26	0.36	0.68	–0.85^*^	–0.92^**^	0.73	–0.92^**^	0.42	0.84^*^
Dy_N_	–0.48	–0.89^**^	–0.81^*^	0.25	0.37	0.68	–0.86^*^	–0.92^**^	0.74	–0.92^**^	0.44	0.83^*^
Ho_N_	–0.49	–0.90^**^	–0.81^*^	0.28	0.37	0.68	–0.87^*^	–0.93^**^	0.76^*^	–0.93^**^	0.41	0.85^*^
Er_N_	–0.47	–0.90^**^	–0.81^*^	0.27	0.38	0.69	–0.87^*^	–0.93^**^	0.75	–0.93^**^	0.42	0.84^*^
Tm_N_	–0.41	–0.91^**^	–0.84^*^	0.26	0.43	0.72	–0.83^*^	–0.92^**^	0.69	–0.91^**^	0.46	0.81^*^
Yb_N_	–0.41	–0.92^**^	–0.85^*^	0.29	0.44	0.73	–0.86^*^	–0.94^**^	0.73	–0.93^**^	0.43	0.84^*^
Lu_N_	–0.35	–0.94^**^	–0.88^**^	0.26	0.48	0.75	–0.84^*^	–0.93^**^	0.71	–0.92^**^	0.47	0.81^*^
(La/Yb)_N_	–0.36	0.89^**^	0.94^**^	–0.26	–0.85^*^	–0.86^*^	0.66	0.70	–0.59	0.67	–0.33	–0.56
(La/Sm)_N_	–0.04	0.93^**^	0.93^**^	–0.52	–0.73	–0.90^**^	0.89^**^	0.89^**^	–.80^*^	0.89^**^	–0.11	–0.85^*^
(Sm/Yb)_N_	–0.48	0.81^*^	0.89^**^	–0.13	–0.84^*^	–0.78^*^	0.53	0.55	–0.47	0.53	–0.39	–0.39
δCe	–0.20	0.37	0.40	–0.58	–0.31	–0.38	0.52	0.32	–0.63	0.44	0.36	–0.51
δEu	0.43	0.83^*^	0.75	–0.11	–0.23	–0.52	0.84^*^	0.73	–0.88^**^	0.824^*^	–0.47	–0.72
δGd	0.35	0.79^*^	0.73	–0.01	–0.35	–0.62	0.84^*^	0.71	–0.87^*^	0.74	–0.41	–0.67

#### Effects of REE of human activities on REE concentrations in SPM

The exceptional REE concentrations of SPM at several specific sites were likely attributed to the intensive influence of human activities. For example, the REEs concentrations at the 1 site were significantly higher than those at the 2 and 3 sites (Table 4), possibly due to river closure by the dam of Wananxi Reservoir. The dam can extend the residence time of upstream water, resulting in the full absorption of REEs by clay mineral particles and organic matter particles, *i.e.,* high REE concentrations of SPM. Generally, river water pH values decrease after flowing across a dam (Wang et al., 2011). A similar phenomenon also occurred in the present study; the river water pH values decreased from 7.58 (at the 1 site) to 6.72 (at the 2 site) (Table 1). As river water pH decreases, many REEs associated particles or colloids are released into dissolved loads (Goldstein & Jacobsen, 1988; Johannesson et al., 2004; Migaszewski, Galuszka & Dolegowska, 2019), resulting in the decrement of REE concentrations of SPM. The significantly higher REE concentrations at the 28 site compared to the nearby sites (Table 4) were likely attributed to agricultural fertilization. The concentrations of major elements at the 28 site did not show anomalous features (Table 2), which can exclude the effects of rock weathering on REE concentrations of SPM. However, the river water NO_3_^−^ concentration at the 28 site was 4 times higher than the average value of Xixi River (Liu & Han, 2020), which was closely associated with the loss of agricultural N. Since the extensive addition of REE within fertilizer (Altomare, Young & Beazley, 2020; Dushyantha et al., 2020; Volokh et al., 1990), agricultural erosion can cause the increment of REE concentrations of SPM. The low REE concentrations at the 34 site compared to the near sites (Table 4) were likely attributed to the strong denudation of regolith. The SPM at the 34 site was characteristic of high Ca and Mg concentrations, low Al and Fe concentrations, and low CIA value (Tables 2 and 3). The result was attributed to the regolith materials which contained low-weathered carbonate minerals were denudated and translocated into river, which explained the low REE concentrations at the site.

### Eeffects of physicochemical properties on REE fractionations of SPM

River SPM is mainly derived from local soils by soil erosion (Linders et al., 2018). However, the PAAS-normalized REE patterns of SPM showed a huge discrepancy compared with local soils (Chen et al., 1992) (Fig. 3). The discrepancy is mainly attributed to REE fractionations between the colloidal (solid) pool and dissolved pool (Elderfield, Upstill-Goddard & Sholkovitz, 1990). Overall, the (La/Yb)_N_ and (Sm/Yb)_N_ ratios of SPM were near 1.5, and the (La/Sm)_N_ ratio was near 1 in the basin (Fig. 4), indicating the enrichments of LREE and MREE relative to HREE in SPM. The enrichment of LREE in SPM is closely linked with the strong adsorption by clay minerals, whereas HREE prefers to form stable soluble complexes (Da Silva et al., 2018). The river water pH plays an important role in affecting the fractionation degree between LREE and HREE in SPM. In the main stream of the Beixi River, the (La/Yb)_N_ ratios of SPM ranged from 1.1 to 2.3 (Fig. 4) and water pH values ranged from 6.7 to 7.6 (Table 1). Similarly, in the granite region of the Zhujiang River basin, the riverine pH decreased from 8.1 to 7.4 during 2000–2014, while the (La/Yb)_N_ ratio of SPM increased from 1.1 to 1.3 (Xu & Han, 2009). Because HREE is preferentially removed from associated SPM into the dissolved load with increasing river water acidity rather than LREE (Migaszewski, Galuszka & Dolegowska, 2019). This reason also explains the increments of (La/Yb)_N_ and (Sm/Yb)_N_ ratios in SPM along the flow direction and negative correlations of them with water pH values in the main stream of the Beixi River (Fig. 4 and Table 6). But the REE fractionation of SPM in the estuary was mainly affected by salinity. The (La/Yb)_N_, (La/Sm)_N_, and (Sm/Yb)_N_ ratios of SPM in the estuary region were significantly positively correlated with TDS and HCO_3_^−^ concentrations (Table 6). Elderfield, Upstill-Goddard & Sholkovitz (1990) reported that the preferential removal of dissolved LREE occurred compared to HREE with the increment of salinity, which meant the relative enrichment of LREE in SPM.

The slight negative Ce anomaly (δCe: 0.8–1.0) of SPM occurred at most sites in the Jiulongjiang River (Fig. 5), likely related to the preferential loss of them compared to other REEs during rock weathering (Smith & Liu, 2018). In addition to weathering process, the negative Ce anomaly of SPM is usually controlled by the water pH value (Elderfield, Upstill-Goddard & Sholkovitz, 1990; Xu & Han, 2009). Dissolved Ce^3+^ is the major form at low pH, but it is easily oxidized to tetravalent Ce with the alkalization of river water (Da Silva et al., 2018). With the increasing water pH value, the removal of dissolved Ce^3+^ as CeO_2_ form leads to Ce enrichment in SPM, *i.e.,* the less negative Ce anomaly of SPM. Thus, the δCe values of SPM positively correlate with riverine pH values (Table 6). However, the correlation relationships between them are not significant (*P* > 0.05) and the variation of δCe values is very slight in the river. These results indicate that the Ce anomaly of SPM is weakly affected by riverine pH, while mainly depends on lithology. Additionally, the intensive negative Ce anomaly occurred at the 1 and 28 sites (Fig. 5), indicating the significant influences of human activities, including the dam effects and agricultural pollutions. Generally, the Eu anomaly of SPM is only controlled by the lithology of the source region (Han et al., 2009). The negative Eu anomaly of SPM occurred at most sites in the Jiulongjiang River which mainly flows through the granite region (Fig. 5), similar results were observed in the Ipojuca River and downstream of Zhujiang River (Da Silva et al., 2018; Xu & Han, 2009). The negative Eu anomaly of SPM in the granite basin mainly depends on the composition and proportion of feldspar minerals, which are commonly Eu-enriched (Nagarajan et al., 2011). However, a positive Eu anomaly of SPM occurred at the 21 and 34 sites where the Ca and Mg concentrations were relatively higher (Table 2 and Fig. 5). Thus, the positive Eu anomaly of SPM at the two sites is mainly attributed to the input of carbonate minerals, which are generally Eu-depleted (Han et al., 2009; Nagarajan et al., 2011). Generally, the positive Gd anomaly of SPM is attributed to the Gd pollution derived from urban wastewater and modern medical treatments (Bau & Dulski, 1996; Nozaki et al., 2000). The δGd values of SPM at most sites in the river were less than 1.6 (Fig. 5), indicating that the Gd in SPM is almost not affected by anthropogenic sources. Additionally, the relatively higher δGd value at the 37 site (1.72) was likely attributed to slight Gd pollution. Gd concentration of SPM at the 37 site was 1.4 times higher, while the concentrations of other REEs were about 1.1 times higher, compared to that at the nearby 36 site. This result indicates that the 37 site is affected by single Gd point pollution. However, the relatively lower δGd value (1.40) at the 28 site was likely associated with agricultural activities. Compared to other sites in the Xixi River, the riverine NO_3_^−^ concentrations at the 28 site were significantly 4 times higher (69 mg/L *vs.* 15 mg/L) (Han et al., 2020; Liu & Han, 2020), indicating strong agricultural activities. Moreover, the concentrations of REEs in SPM at the 28 site were about 3–4 times higher than those at the other sites in the Xixi River, indicating the anthropogenic input of REEs from agricultural soils. Overall, the 28 site is affected by agricultural Gd pollution although the δGd value is less than 1.6.

## Conclusions

The concentrations and fractionations of REEs in SPM were investigated in rivers regions (including the main stream and tributary of Beixi River, Xixi River, Nanxi River, and estuary) of the Jiulongjiang River. There were similar REE concentrations of SPM in the main stream of Beixi River, Xixi River, and Nanxi River, these mainly flow through the granite region; while it was lower in the tributary of Beixi River, which is associated with widely distributed carbonates. However, the lower REE concentrations of SPM in the estuary are mainly attributed to the removal of REEs with the increasing salinity. Overall, the REE concentrations of SPM in riverine water are primarily controlled by lithologic distribution, while it is also affected by salinity in the estuary. Riverine pH plays an important role in affecting REE fractionation of SPM in the river, but the fractionation is mainly controlled by salinity in the estuary. These results indicate that the increasing salinity from the inland river to the estuary affects the concentrations and fractionations of REEs in SPM.

## Supplemental Information

10.7717/peerj.12414/supp-1 Supplemental Information 1 Raw measurements

## Additional Information and Declarations

### Data Availability

The following information was supplied regarding data availability: The raw measurements are available in the Supplementary File.

## References

[ref-1] Alderton DHM, Pearce JA, Potts PJ (1980). Rare earth element mobility during granite alteration: evidence from southwest England. Earth and Planetary Science Letters.

[ref-2] Altomare AJ, Young NA, Beazley MJ (2020). A preliminary survey of anthropogenic gadolinium in water and sediment of a constructed wetland. Journal of Environmental Management.

[ref-3] Amalan K, Ratnayake AS, Ratnayake NP, Weththasinghe SM, Dushyantha N, Lakmali N, Premasiri R (2018). Influence of nearshore sediment dynamics on the distribution of heavy mineral placer deposits in Sri Lanka. Environmental Earth Sciences.

[ref-4] Bau M, Dulski P (1996). Anthropogenic origin of positive gadolinium anomalies in river waters. Earth and Planetary Science Letters.

[ref-5] Bayon G, Toucanne S, Skonieczny C, Andre L, Bermell S, Cheron S, Dennielou B, Etoubleau J, Freslon N, Gauchery T, Germain Y, Jorry SJ, Ménot G, Monin L, Ponzevera E, Rouget ML, Tachikawa K, Barrat JA (2015). Rare earth elements and neodymium isotopes in world river sediments revisited. Geochimica Et Cosmochimica Acta.

[ref-6] Brookins DG (1989). Aqueous geochemistry of rare-earth elements. Reviews in Mineralogy.

[ref-7] Chelnokov GA, Bragin IV, Kharitonova NA (2020). Geochemistry of rare earth elements in the rivers and groundwaters of chistovodnoe thermal area (Primorye, Far East of Russia). IOP Conference Series: Earth and Environmental Science.

[ref-8] Chen Z, Chen C, Liu Y, Wu Y, Yang S, Lu C (1992). Study on soil environmental background values in Fujian Province. Chinese Journal of Environmental Science.

[ref-9] Cholet C, Steinmann M, Charlier J-B, Denimal S (2019). Characterizing fluxes of trace metals related to dissolved and suspended matter during a storm event: application to a karst aquifer using trace metals and rare earth elements as provenance indicators. Hydrogeology Journal.

[ref-10] Dagg M, Benner R, Lohrenz S, Lawrence D (2004). Transformation of dissolved and particulate materials on continental shelves influenced by large rivers: plume processes. Continental Shelf Research.

[ref-11] Da Silva Y, Do Nascimento C, Da Silva Y, Amorim F, Cantalice J, Singh V, Collins A (2018). Bed and suspended sediment-associated rare earth element concentrations and fluxes in a polluted Brazilian river system. Environmental Science and Pollution Research.

[ref-12] Dushyantha N, Batapola N, Ilankoon IMSK, Rohitha S, Premasiri R, Abeysinghe B, Ratnayake N, Dissanayake K (2020). The story of rare earth elements (REEs): occurrences, global distribution, genesis, geology, mineralogy and global production. Ore Geology Reviews.

[ref-13] Elderfield H, Upstill-Goddard R, Sholkovitz ER (1990). The rare earth elements in rivers, estuaries, and coastal seas and their significance to the composition of ocean waters. Geochimica Et Cosmochimica Acta.

[ref-14] Elias MS, Ibrahim S, Samuding K, Kantasamy N, Rahman SA, Hashim A (2019). Rare earth elements (REEs) as pollution indicator in sediment of Linggi River, Malaysia. Applied Radiation and Isotopes.

[ref-15] Goldstein SJ, Jacobsen SB (1988). Rare-earth elements in river waters. Earth and Planetary Science Letters.

[ref-16] Han G, Tang Y, Liu M, Van Zwieten L, Yang X, Yu C, Wang H, Song Z (2020). Carbon-nitrogen isotope coupling of soil organic matter in a karst region under land use change, Southwest China. Agriculture, Ecosystems and Environment.

[ref-17] Han G, Xu Z, Tang Y, Zhang G (2009). Rare earth element patterns in the Karst Terrains of Guizhou Province, China: implication for water/particle interaction. Aquatic Geochemistry.

[ref-18] Han G, Yang K, Zeng J (2021). Distribution and fractionation of rare earth elements in suspended sediment of the Zhujiang River, Southwest China. Journal of Soils and Sediments.

[ref-19] Hissler C, Hostache R, Iffly JF, Pfister L, Stille P (2015). Anthropogenic rare earth element fluxes into floodplains: coupling between geochemical monitoring and hydrodynamic sediment transport modelling. Comptes Rendus Geoscience.

[ref-20] Huang J, Zhang Z, Feng Y, Hong H (2013). Hydrologic response to climate change and human activities in a subtropical coastal watershed of southeast China. Regional Environmental Change.

[ref-21] Jackson GA, Burd AB (2015). Simulating aggregate dynamics in ocean biogeochemical models. Progress in Oceanography.

[ref-22] Johannesson KH, Tang J, Daniels JM, Bounds WJ, Burdige DJ (2004). Rare earth element concentrations and speciation in organic-rich blackwaters of the Great Dismal Swamp, Virginia, USA. Chemical Geology.

[ref-23] Jones AM, Xue Y, Kinsela AS, Wilcken KM, Collins RN (2016). Donnan membrane speciation of Al, Fe, trace metals and REEs in coastal lowland acid sulfate soil-impacted drainage waters. Science of the Total Environment.

[ref-24] Krickov IV, Lim AG, Manasypov RM, Loiko SV, Vorobyev SN, Shevchenko VP, Dara OM, Gordeev VV, Pokrovsky OS (2020). Major and trace elements in suspended matter of western Siberian rivers: first assessment across permafrost zones and landscape parameters of watersheds. Geochimica Et Cosmochimica Acta.

[ref-25] Li X, Han G (2021). One-step chromatographic purification of K, Ca, and Sr from geological samples for high precision stable and radiogenic isotope analysis by MC-ICP-MS. Journal of Analytical Atomic Spectrometry.

[ref-26] Li X, Han G, Zhang Q, Miao Z (2020). An optimal separation method for high-precision K isotope analysis by using MC-ICP-MS with a dummy bucket. Journal of Analytical Atomic Spectrometry.

[ref-27] Li Z, Qiu J-S, Yang X-M (2014). A review of the geochronology and geochemistry of Late Yanshanian (Cretaceous) plutons along the Fujian coastal area of southeastern China: implications for magma evolution related to slab break-off and rollback in the Cretaceous. Earth-Science Reviews.

[ref-28] Linders T, Infantes E, Joyce A, Karlsson T, Ploug H, Hassellöv M, Sköld M, Zetsche E-M (2018). Particle sources and transport in stratified Nordic coastal seas in the Anthropocene. Elementa: Science of the Anthropocene.

[ref-29] Liu J, Han G (2020). Major ions and δ34SSO4 in Jiulongjiang River water: investigating the relationships between natural chemical weathering and human perturbations. Science of the Total Environment.

[ref-30] Liu J, Han G (2021). Tracing riverine particulate black carbon sources in Xijiang River Basin: insight from stable isotopic composition and bayesian mixing model. Water Research.

[ref-31] Liu M, Han G, Li X (2021). Comparative analysis of soil nutrients under different land-use types in the Mun River basin of Northeast Thailand. Journal of Soils and Sediments.

[ref-32] Louis P, Messaoudene A, Jrad H, Abdoul-Hamid BA, Vignati DAL, Pons MN (2020). Understanding Rare Earth Elements concentrations, anomalies and fluxes at the river basin scale: the Moselle River (France) as a case study. Science of the Total Environment.

[ref-33] Louvat P, Allègre CJ (1998). Riverine erosion rates on Sao Miguel volcanic island, Azores archipelago. Chemical Geology.

[ref-34] Ma L, Dang DH, Wang W, Evans RD, Wang W-X (2019). Rare earth elements in the Pearl River Delta of China: potential impacts of the REE industry on water, suspended particles and oysters. Environmental Pollution.

[ref-35] McLennan SM (1993). Weathering and global denudation. The Journal of Geology.

[ref-36] Michaelides K, Ibraim I, Nord G, Esteves M (2010). Tracing sediment redistribution across a break in slope using rare earth elements. Earth Surface Processes and LandForms.

[ref-37] Migaszewski ZM, Galuszka A (2015). The characteristics, occurrence, and geochemical behavior of rare earth elements in the environment: a review. Critical Reviews in Environmental Science and Technology.

[ref-38] Migaszewski ZM, Gałuszka A, Dołęgowska S (2019). Extreme enrichment of arsenic and rare earth elements in acid mine drainage: case study of Wiśniówka mining area (south-central Poland). Environmental Pollution.

[ref-39] Naccarato A, Tassone A, Cavaliere F, Elliani R, Pirrone N, Sprovieri F, Tagarelli A, Giglio A (2020). Agrochemical treatments as a source of heavy metals and rare earth elements in agricultural soils and bioaccumulation in ground beetles. Science of the Total Environment.

[ref-40] Nagarajan R, Madhavaraju J, Armstrong-Altrin JS, Nagendra R (2011). Geochemistry of Neoproterozoic limestones of the Shahabad Formation, Bhima Basin, Karnataka, southern India. Geosciences Journal.

[ref-41] Nesbitt HW, Young GM (1982). Early Proterozoic climates and plate motions inferred from major element chemistry of lutites. Nature.

[ref-42] Nozaki Y, Lerche D, Alibo DS, Snidvongs A (2000). The estuarine geochemistry of rare earth elements and indium in the Chao Phraya River, Thailand. Geochimica Et Cosmochimica Acta.

[ref-43] Olivarez AM, Owen RM (1991). The europium anomaly of seawater: implications for fluvial versus hydrothermal REE inputs to the oceans. Chemical Geology.

[ref-44] Petelet-Giraud E, Klaver G, Negrel P (2009). Natural versus anthropogenic sources in the surface- and groundwater dissolved load of the Dommel river (Meuse basin): constraints by boron and strontium isotopes and gadolinium anomaly. Journal of Hydrology.

[ref-45] Quinn KA, Byrne RH, Schijf J (2006). Sorption of yttrium and rare earth elements by amorphous ferric hydroxide: influence of solution complexation with carbonate. Geochimica Et Cosmochimica Acta.

[ref-46] Rogers KL, Bosman SH, Weber S, Magen C, Montoya JP, Chanton JP (2019). Sources of carbon to suspended particulate organic matter in the northern Gulf of Mexico. Elementa-Science of the Anthropocene.

[ref-47] Roussiez V, Aubert D, Heussner S (2013). Continental sources of particles escaping the Gulf of Lion evidenced by rare earth elements: flood vs. normal conditions. Marine Chemistry.

[ref-48] Shabani MB, Masuda A (1991). Sample introduction by on-line two-stage solvent extraction and back-extraction to eliminate matrix interference and to enhance sensitivity in the determination of rare earth elements with inductively coupled plasma mass spectrometry. Analytical Chemistry.

[ref-49] Shajib MTI, Hansen HCB, Liang T, Holm PE (2020). Rare earth elements in surface specific urban runoff in Northern Beijing. Science of the Total Environment.

[ref-50] Sholkovitz ER, Landing WM, Lewis BL (1994). Ocean particle chemistry-The fractionation of rare-earth elements between suspended particles and seawater. Geochimica Et Cosmochimica Acta.

[ref-51] Smith C, Liu X-M (2018). Spatial and temporal distribution of rare earth elements in the Neuse River, North Carolina. Chemical Geology.

[ref-52] Suja S, Fernandes LL, Rao VP (2017). Distribution and fractionation of rare earth elements and Yttrium in suspended and bottom sediments of the Kali estuary, western India. Environmental Earth Sciences.

[ref-53] Taylor SR, McLennan SM (1985). The continental crust: its composition and evolution.

[ref-54] Vercruysse K, Grabowski RC, Rickson RJ (2017). Suspended sediment transport dynamics in rivers: multi-scale drivers of temporal variation. Earth-Science Reviews.

[ref-55] Viers J, Dupre B, Gaillardet J (2009). Chemical composition of suspended sediments in World Rivers: new insights from a new database. Science of the Total Environment.

[ref-56] Volokh AA, Gorbunov AV, Gundorina SF, Revich BA, Frontasyeva MV, Chen Sen P (1990). Phosphorus fertilizer production as a source of rare-earth elements pollution of the environment. Science of the Total Environment.

[ref-57] Wang F, Wang B, Liu C-Q, Wang Y, Guan J, Liu X, Yu Y (2011). Carbon dioxide emission from surface water in cascade reservoirs–river system on the Maotiao River, southwest of China. Atmospheric Environment.

[ref-58] WRB IWG (2014). World Reference Base for soil resources 2014: international soil classification system for naming soils and creating legends for soil maps. World Soil Resources Report.

[ref-59] Xu Z, Han G (2009). Rare earth elements (REE) of dissolved and suspended loads in the Xijiang River, South China. Applied Geochemistry.

[ref-60] Yang K, Han G, Zeng J, Zhou W (2021). Distribution, fractionation and sources of rare earth elements in suspended particulate matter in a tropical agricultural catchment, northeast Thailand. PeerJ.

[ref-61] Zeng J, Han G (2020). Preliminary copper isotope study on particulate matter in Zhujiang River, southwest China: application for source identification. Ecotoxicology and Environmental Safety.

[ref-62] Zeng J, Han G, Yang K (2020). Assessment and sources of heavy metals in suspended particulate matter in a tropical catchment, Northeast Thailand. Journal of Cleaner Production.

